# Dyadic reports of weight control practices, sedentary behaviors, and family functioning and communication between adult weight management patients and their children

**DOI:** 10.1002/osp4.481

**Published:** 2021-02-07

**Authors:** Pratt Keeley J., VanFossen Catherine A., Kiser Haley M., Whiting Riley, Spees Colleen, Taylor Chris A., Eneli Ihuoma, Noria Sabrena

**Affiliations:** ^1^ Department of Human Sciences Human Development and Family Science Program College of Education and Human Ecology The Ohio State University Columbus Ohio USA; ^2^ Department of Surgery The Ohio State Wexner Medical Center Columbus Ohio USA; ^3^ Department of Pediatrics The Ohio State University Columbus Ohio USA; ^4^ Divison of Medical Dietetics School of Health and Rehabilitation Sciences The Ohio State University College of Medicine Columbus Ohio USA

**Keywords:** family functioning, parent, sedentary activity, weight control practices, weight management

## Abstract

**Background:**

Parents are increasingly pursuing weight loss in medical weight management programs, yet little is known about the presenting behaviors and practices of children who have parents initiating these programs.

**Objective:**

To describe congruence in weight control practices, sedentary and screen time behaviors, and family functioning and communication between parents initiating a medical weight management program and their children (ages 7–18).

**Methods:**

Twenty‐three dyads were enrolled and had measured height/weight and research packets completed including perceived weight status, weight control practices, sedentary and screen‐time behaviors, and family functioning and communication. Paired *t*‐tests and intraclass correlations assessed congruence; independent *t*‐tests determined differences based on child demographics (age, sex, and weight status).

**Results:**

Parents underestimated children's use of weight control practices compared to child reports. Children with overweight, males, and older in age had increased weight control practices and sedentary and screen‐time behaviors. Children who perceived themselves to have overweight reported more impaired family communication than children perceived to be a healthy weight.

**Conclusions:**

This study highlights the discrepancy between dyads' reports of children's behaviors, and identifies that specific child populations with overweight, older in age, and males are at‐risk of experiencing less healthy behaviors and impaired family communication. Future research should monitor changes over time in parental weight management programs to determine effects based on parental weight loss.

## 1 INTRODUCTION

Adult obesity rates have steadily increased for the past 3 decades,^1^ ^,^ ^2^ with half of all US adults pursuing weight loss, increasingly through medical weight management programs.^3^ ^,4^ Given their shared genetics and children's exposure to parental behaviors in the home contributing to parental obesity,^5^ children of parents in weight management programs are a particularly high‐risk group for developing obesity.^6^, ^7^, ^8^, ^9^, ^10^, ^11^ Prior work has documented that parents in weight management programs and who had bariatric surgery report their older children and children with obesity engage in higher rates of weight control practices (i.e., dieting),^9^ and more frequent talk about child weight and parental weight loss.^10^ ^,^ ^12^ Additionally, parents report more impaired family functioning when their children have overweight/obesity.^6^,^7^ However, a limitation of prior research with children of parents in weight management programs is the assessment of parent‐only perspectives of children's behaviors.^6^ ^,^ ^7^ ^,^ ^9^, ^10^, ^11^, ^12^

Parents and children often have different perceptions about their own behaviors and the behaviors of the other member of the dyad.^13^ ^,^ ^14^ Lack of congruence between parents and children challenges researchers to understand the accuracy of individual assessments of behaviors or practices.^15^ Additionally, lack of congruence in the assessment of relationship factors, may indicate problems in which one member of the dyad believes their relationship is functional while the other believes it is impaired.^16^ ^,^ ^17^ Prospective assessment of parent–child dyads, in which both parent and child perceptions are obtained, are needed to understand significant factors to assess over parental weight management program duration that may be modifiable in future parent–child interventions.

The objective of this study was to describe the dyadic reports of weight control practices, sedentary and screen time behaviors, and family functioning and communication between parent–child dyads in which the parent is initiating an adult medical weight management programs. Agreement or congruence between parental and children's reports was explored, and between group differences based on child demographics (age, sex, and weight status) was conducted. Given the prior cross‐sectional evidence on parents in weight management programs,^6^ ^,^ ^7^ ^,^ ^10^, ^11^, ^12^ following hypotheses were assessed: (1) parents of children and children with overweight/obesity will report more impaired family functioning, and (2) parents of older children and children with overweight/obesity will report higher use of weight control practices compared to younger children and children with a healthy weight status.

## 2 MATERIALS AND METHODS

### 2.1 Recruitment and enrollment

Recruitment took place using a convenience sampling strategy from November 2018 to March 2020 at parents' weight management program orientation meeting. Details about The Ohio State University (OSU) Center for comprehensive weight management, metabolic and bariatric surgery have been described previously.^18^ The 6 month program includes nutrition, exercise, and behavioral components delivered through group educational and support classes and individual consultations with registered dietitians, behavioral health providers, and exercise physiologists. There is an initial wellness orientation, in which patients have their resting metabolic rate tested and a fitness evaluation to formulate individualized meal and exercise plans, respectively. Weight, dietary, and exercise journals are reviewed weekly with a postprogram fitness evaluation. Inclusion criteria comprised: parent enrolled in the weight management program, child aged 7–18 years old living in the home ≥4 days per week with the parent, no history of bariatric surgeries, no life‐threatening comorbidities for the parent or child (i.e., terminal cancer), and parent and child need to be free of conditions that would prevent engagement in physical activity (i.e., unable engage in movement‐based physical activity). The child age range of 7–18 was selected due to appropriateness of child self‐report measures. If multiple children met inclusion criteria, parents were encouraged to select their youngest child. Parents who indicated interest at their orientation were provided contact information to schedule with their child, in which consent/assent were obtained and dyads were enrolled. Parents and children completed a research packet and had height and weight measured. Children were offered support from a member of the research team if they needed assistance with completing the measures. Parents and children received a $20 retail gift card for participation. The study received OSU Institutional Review Board approval (IRB #2018H0308).

### 2.2 Measures

Based on the measured height and weight using a wall‐mounted stadiometer and a scale^19^ and child date of birth, BMI and child BMI percentile was calculated, and weight status categories were made.^20^ ^,^ ^21^ Parents and children each provided their perception of children's weight status: “Right now, do you consider your/your child's weight status: underweight, healthy weight, overweight, obese.”

*Parent and child weight control practices* were used from Project Eating and Activity over Time (EAT),^22^ in which parents responded yes (1) or no (0) to practices utilized for their child and themselves in the past year. Children responded to the same questions for themselves. The question stem was: ‘‘Have you [or has your child] done any of the following things in order to lose weight or keep from gaining weight during the past year?’’ Weight control practices were categorized into 10 unhealthy (fasted, dieted, ate very little food, used a food substitute, skipped meals, took diet pills, used laxatives, used diuretics, smoked more cigarettes, made myself vomit) and five healthy (increased fruit and vegetables, cut out between meal snacking, exercised, decreased fat intake, reduced calorie intake) practices. Combining unhealthy and health weight control practices, the total score possible for all weight control practices was 15; 10 for unhealthy and five for healthy weight control practices. Higher scores indicate higher use.

*Children's sedentary and screen‐time behaviors* were assessed from Project EAT^22^ with the following stem for each question, “In your (or your child's) free time on an average weekday (or weekend), how many hours do you (do they) spend doing the following activities?” Activities included watching TV, using a computer, playing video games (both interactive and sitting), and mobile device use. Parents and children responded with, “none, less and ½ hour a week, 1/2 h to 2 h a week, 2 to 4 h a week, 4 ½ to 6 h a week, or 6+ hours a week.” A total of score weekday/weekend use was calculated for parent and child reports of children's total sedentary behaviors and individual totals TV, computer, videogame and interactive videogame, and mobile use. Higher scores indicate higher weekly utilization.

#### 2.2.1 Family functioning and communication

The *Family Assessment Device‐General Functioning Scale* was completed by parents and children to measure current family functioning.^23^ A clinical cut‐off score was used to note clinically impaired (≥2.0) or healthy family functioning (<2.0).^15^ ^,^ ^23^ *Fmily Assessment Device‐Communication Scale* was used to assess the clarity and directness of communication between family members.^23^ The clinical cut‐off score notes clinically impaired (≥2.2) or healthy family communication (<2.2).^20^ Higher scores on both scales indicate more impairment. These scales have been previously validated with school age children and adolescents; however, children younger than 12 years old had slightly lower reliability but concurrent validity with parent report.^24^

### 2.3 Analysis

Data were analyzed using SPSS version 27 (IBM). Descriptive statistics were conducted for all scales. Paired *t*‐tests and intraclass correlations (ICCs) determined congruence between child and parent reports. Independent *t*‐tests assessed differences by child demographic characteristics (age group [younger children 7–11, older children 12–18], sex [male, female], objectively measured weight status [healthy weight, overweight/obese], and perceived weight status [healthy weight, overweight]). Significance was determined by *p* ≤ 0.05.

## 3 RESULTS

Fifty‐three parents with children who met inclusion criteria were invited to participate. Of the 53, 23 (43.3%) dyads provided consent/assent and completed the data collection. Of the 30 (56.7%) who did not agree to participate, 10 parents indicated initial interest at their orientation but did not return follow‐up calls. Of the other 20, eight declined due to lack of interest from child, six due to child time constraints, and six declined to give a reason. Demographic characteristics and scale descriptives are in Tables 1 and 2, respectively.

**TABLE 1:** Parent, child, and household demographics and scale descriptives (*N* = 23) [% (*n*) or mean ± SD]

Parent		Child	
Sex		Sex	
Female	78.3% (18)	Female	52.2% (12)
Male	21.7% (5)	Male	47.8% (11)
Race		Race	
White	73.9% (17)	White	65.2% (15)
African American/Black	17.4% (4)	African American/Black	17.4% (4)
Asian	8.7% (2)	Multiracial	8.7% (2)
		Other	8.7% (2)
Ethnicity		Ethnicity	
Hispanic	0	Hispanic	17.4% (4)
Not Hispanic	100% (23)	Not Hispanic	82.6% (19)
Age	43.39 ± 5.74	Age	12.3 ± 3.27
BMI	43.74 ± 8.68	BMI	23.16 ± 6.51
Weight status		** ** Weight status	
Class I obesity	13% (3)	Healthy weight	52.2% (12)
Class II obesity	34.8% (8)	Overweight	13.0% (3)
Class III obesity	52.2% (12)	Obese	34.8 (8)
Perceived parent weight status by parent		Perceived child weight status by parent	
Overweight	21.7% (5)	Underweight	4.3% (1)
Obese	78.3% (18)	Healthy weight	65.2% (15)
Education		Overweight	30.4% (7)
High school graduate	4.3% (1)	Perceived child weight status by child	
Associate degree	8.7% (2)	Underweight	26.1% (6)
Bachelor's degree	69.6% (16)	Healthy weight	47.8% (11)
Master's degree or higher	17.4% (4)	Overweight	26.1% (6)
		BMI percentile	73.35 ± 26.39
Household			
Annual household income		** ** Relationship status	
$40,000–59,999	17.4% (4)	Married	69.6% (16)
$60,000–99,000	34.8% (8)	Divorced	17.4% (4)
$100,000+	47.8% (11)	Single	8.4% (2)
Number of children	2 ± 1.13	Perceived partner weight status	
Food security		Healthy weight	26.1% (6)
Secure	95.7% (22)	Overweight	39.1% (9)
Insecure	4.3% (1)	Obese	17.4% (4)

**TABLE 2:** Descriptive statistics for scales and subscales

		Mean ± SD	Range	Cronbach's alpha
Weight control practices	Total parent about parent	6.35 ± 2.9	0.00–11.00	0.75
	Total parent about child	1.35 ± 2.06	0.00–6.00	0.78
	Total child about child	2.57 ± 2.57	0.00–8.00	0.78
	Unhealthy parent about parent	2.91 ± 1.83	0.00–6.00	0.61
	Unhealthy parent about child	0.39 ± 0.78	0.00–3.00	0.50
	Unhealthy child about child	0.74 ± 1.29	0.00–4.00	0.60
	Healthy parent about parent	3.43 ± 1.67	0.00–5.00	0.79
	Healthy parent about child	0.96 ± 1.61	0.00–5.00	0.86
	Healthy child about child	1.83 ± 1.53	0.00–4.00	0.77
Sedentary and screen time behaviors	Sedentary week/end total parent about child	73.5 ± 20.52	40.00–117.00	0.71
	Sedentary week/end total child about child	66.96 ± 4.03	25.00–126.00	0.61
	TV total parent about child	31.35 ± 0.49	16.00–49.00	0.65
	TV total child about child	26.83 ± 9.25	11.00–47.00	0.45
	Computer total parent about child	23.73 ± 2.91	7.00–49.00	0.69
	Computer total child about child	21.91 ± 0.54	7.00–49.00	0.68
	Videogames total parent about child	19.61 ± 2.42	7.00–42.00	0.90
	Videogames total child about child	18.22 ± 2.25	7.00–49.00	0.90
	Mobile total parent about child	33.09 ± 14.4	7.00–49.00	0.94
	Mobile total child about child	31.7 ± 15.41	7.00–49.00	0.96
	Interactive video game total parent about child	6.91 ± 0.42	5.00–7.00	0.73
	Interactive video game total child about child	9.22 ± 5.4	7.00–28.00	0.91
Family functioning and communication	Family communication parent	1.89 ± 0.38	1.00–2.44	0.66
	General functioning parent	1.7 ± 0.46	1.00–2.83	0.85
	Family communication child	2.14 ± 0.48	1.44–3.33	0.67
	General functioning child	1.79 ± 0.59	1.00–3.75	0.89

### 3.1 Congruence between parent and child parallel reports

#### 3.1.1 Perception of Children's weight status

Children were split between a healthy weight status (52%) and overweight or obesity (48%) based on objective measurements. Parents and children primarily perceived children's weight status to be a “healthy weight” (70% and 74%, respectively). The majority of children were accurate in perceiving their weight status (19, 83%), four children had inaccurate perceptions (two, 9% underestimated and two, 9% overestimated). The majority of parents (18, 78%) also accurately perceived their child's weight status, with five (22%) underestimating their child's weight status.

#### 3.1.2 Weight control practices

Parents' self‐reports of their total weight control practices were significantly higher than their reports of their children's total weight control practices (*p* < 0.001), and their children's self‐reports (*p* < 0.001). However, parents reports of their children's weight control practices were lower than children's self‐reports (*p* = 0.019). These results were consistent for healthy and unhealthy practices (see Table 3). There was moderate inter‐rater agreement across these scales (ICC = 0.54–0.65).

**TABLE 3:** Parent and child congruence on weight control practices and Children's sedentary and screen‐time behaviors

		Mean	SD	SE	Lower 95% CI	Upper 95% CI	t	*df*	Sig. (2‐tailed)	Intraclass correlations	
										*r*	ICC
Total	Parent self‐report × child self‐report	3.78	4.00	0.83	2.05	5.51	4.54	22	0.00^b^	–	–
	Parent self‐report × parent report of child	5.00	2.97	0.62	3.72	6.28	8.08	22	0.00^b^	–	–
	Parent report of child × child self‐report	–1.22	2.32	0.48	–2.22	–0.22	–2.52	22	0.02^a^	0.52	0.63^b^
Healthy	Parent self‐report × child self‐report	1.61	2.37	0.49	0.58	2.63	3.26	22	0.00^b^	–	–
	Parent self‐report × parent report of child	2.48	1.78	0.37	1.71	3.25	6.68	22	0.00^b^	–	–
	Parent report of child × child self‐report	–0.87	1.49	0.31	–1.51	–0.23	–2.81	22	0.01^a^	0.55	0.65^b^
Unhealthy	Parent self‐report × child self‐report	2.17	2.04	0.42	1.29	3.05	5.12	22	0.00^b^	–	–
	Parent self‐report × parent report of child	2.52	1.73	0.36	1.77	3.27	7.00	22	0.00^b^	–	–
	Parent report of child × child self‐report	–0.35	1.19	0.25	–0.86	0.17	–1.40	22	0.18	0.42	0.54^a^
Sedentary & screen‐time behaviors	Sedentary behavior parent report of child × child self‐report	6.86	24.85	5.30	–4.15	17.88	1.30	21	0.21	0.40	0.56^a^
	TV parent report of child × child self‐report	4.52	12.41	2.59	–0.84	9.89	1.75	22	0.09	0.22	0.33
	Computer parent report of child × child self‐report	1.77	15.47	3.30	–5.09	8.63	0.54	21	0.60	0.16	0.27
	Video games parent report of child × child self‐report	1.39	11.41	2.38	–3.54	6.33	0.59	22	0.57	0.57^b^	0.73^b^
	Mobile/Tablet parent report of child × child self‐report	1.39	12.82	2.67	–4.15	6.93	0.52	22	0.61	0.63^b^	0.78^b^
	Interactive video games parent report of child × child self‐report	–2.30	5.38	1.12	–4.63	0.02	–2.05	22	0.05^a^	0.09	0.02

^a^ Significant at the 0.05 level (2‐tailed).

^b^ Significant at the 0.01 level (2‐tailed).

#### 3.1.3 Sedentary and screen‐time behaviors

Although not significant, parents reported that their children had higher utilization rates of sedentary and screen‐time behaviors than children self‐reported (see Table 3). There was one exception for interactive video games, in which parents reported significantly lower child use than children self‐reported (*p* = 0.05). Inter‐rater agreement ranged from low to good across these scales (ICC = 0.27–0.78).

#### 3.1.4 Family functioning

Parents (1.70 ± 0.46) and children (1.79 ± 0.53; *t*(22) = ‐0.67, *p* = 0.513) had similar reports of family functioning. However, parents reported significantly better family communication (1.89 ± 0.38) compared to children's reports (2.14 ± 0.48; *t*(22) = ‐2.32, *p* = 0.030). Six parents (26.1%) and six children (26.1%) reported clinically impaired family functioning, while six parents (26.1%) and 11 children (47.8%) reported clinically impaired family communication.

### 3.2 Differences based on child demographic characteristics

#### 3.2.1 Child age

Older children (1.23 ± 1.48) self‐reported more unhealthy weight control practices compared to younger children (0.10 ± 0.47; *t*(16.23) = ‐2.53, *p* = 0.022). Older children (77.62 ± 22.11) also self‐reported higher total sedentary behaviors than younger children (53.10 ± 19.56; *t*(21) = ‐2.77, *p* = 0.012). This was consistent for older children with computer (older 26.62 ± 10.70, younger 15.80 ± 6.68, *t*(21) = ‐2.79, *p* = 0.011), video game (older 23.08 ± 13.00; younger 11.90 ± 7.51, *t*(21) = ‐2.39, *p* = 0.026), and mobile/table use (older 37.54 ± 11.76, younger 24.10 ± 16.81, *t*(21) = ‐2.26, *p* = 0.035). Parents also reported that their older children (41.08 ± 8.62) had higher mobile/tablet use compared to younger children (22.70 ± 13.99; *t*(21) = ‐3.89, *p* = 0.001).

#### 3.2.2 Child sex

Male children (*n* = 11; 77.72 ± 24.76) self‐reported higher total sedentary behaviors compared to female children (*n* = 12; 57.83 ± 19.38; *t*(21) = ‐2.24, *p* = 0.036). Male children (24.36 ± 13.50) and their parents (27.09 ± 12.48) reported higher use of video games compared to female children (12.58 ± 7.89; *t*(21) = ‐2.58, *p* = 0.017) and their parents (12.75 ± 7.71; *t*(21) = ‐3.35, *p* = 0.003).

#### 3.2.3 Child weight status

Children with a measured healthy weight status (*n* = 12; 1.17 ± 1.34) self‐reported using less healthy weight control practices compared to children with overweight/obesity (*n* = 11; 2.55 ± 1.44; *t*(21) = ‐2.38, *p* = 0.027). Conversely, parents of children with a measured healthy weight status (4.08 ± 1.28) self‐reported higher utilization of healthy weight control practices compared to parents of children with overweight/obesity (2.73 ± 1.74; *t*(21) = 2.08, *p* = 0.053). Children who perceived their weight status to be overweight (*n* = 6) reported higher utilization of total weight control practices (overweight 4.83 ± 2.32, healthy 1.76 ± 22.19, *t*(21) = ‐2.91, *p* = 0.008), healthy weight control practices (overweight 3.00 ± 0.1.26, healthy 1.41 ± 1.42; *t*(21) = ‐2.42, *p* = 0.025), and unhealthy weight control practices (overweight 1.83 ± 0.1.33, healthy 0.35 ± 1.06; *t*(21) = ‐2.76, *p* = 0.021) compared to children who perceived their weight status to be healthy (*n* = 17). Children who perceived their weight status to be overweight (2.57 ± 0.53) reported more impaired family communication than children who perceived their weight status to be healthy (1.98 ± 0.36; *t*(21) = ‐3.02, *p* = 0.005).

## 4 DISCUSSION

This appears to be the first study to enroll parents and their children when a parent is initiating a medical weight management program to determine the congruence between parent and children reports in weight control practices, sedentary and screen time behaviors, and family functioning and communication. By comparing agreement between parental and child reports, it was identified that parents underestimated their children's use of weight control practices. Based on the child demographics, children perceived to have overweight/obesity reported using more weight control practices and reported more impaired family communication. Additionally, older children reported higher use of weight control practices, and older children and male children had increased sedentary and screen‐time behaviors. The prevalence of overweight/obesity that parents perceived their children to have in this study (30%), was similar to prior reports of parents' perceptions of their child's weight status (44%^7^; 23%^6^). In this study 22% of parents underestimated their child's weight status. Parental underestimation of children's obesity is well documented,^25^, ^26^, ^27^ but not among parents who are actively seeking weight loss in weight management programs. Parents were more likely to underestimate their child's weight status than children were to underestimate their own weight status (22% vs. 9%). Future assessment of the effects of parental weight loss in weight management programs on perceptions of and changes in actual child weight status will provide objective means of determining if children of parents in weight management programs loss or gain weight at unhealthy rates, and use unhealthy means of weight control, during parental program participation.

Parents underestimated their child's weight control practices. In prior work utilizing parent‐only reports, Pratt et al.^9^ found that parents in weight management programs and who had bariatric surgery reported that their older children and children with obesity were more likely to use weight control practices. Similarly, in this study, but based on child reports, older children reported using more unhealthy weight control practices, and children with overweight/obesity reported using more healthy weight control practices. Children who perceived their weight status to be overweight/obese reported greater total, healthy, and unhealthy weight control practices compared to children who perceived themselves to be a healthy weight. Developmentally, older children are more likely to be aware of their parents' behaviors and practices related to weight loss, and are exposed to more social pressure to be slim or a healthy weight, and children with overweight/obesity experience increased pressure to reduce their weight.^28^ It is essential for future research to discern how parental participation and subsequent weight loss in weight management programs affects children's weight control practices to determine if these children are placed at greater risk of developing disordered eating behaviors or eating disorders, especially among older children and those with obesity.

Parents overestimated sedentary and screen‐time behaviors that their child participated in, with the significant exception of video game use. Older children and male children reported significantly higher sedentary behaviors and older children had significantly higher reports of every screen‐time behavior. Male children and their parents both reported significantly higher video game use compared to female children. Future research should include assessments of parental and child physical activity to determine if higher rates of children's sedentary and screen time behaviors are correlated with lower parental and child physical activity frequency.

The rate of impaired family functioning in this study (26%) was similar to reports of parents in weight management programs or pursuing bariatric surgery (45%^7^; 25%^6^), but high compared to most child populations and those in pediatric primary care (i.e., 13%).^29,^ ^30^ Children reported significantly more impaired family communication compared to parents, especially for children who perceived their weight status to be overweight/obese. Based on the parental reports, Pratt et al.^7^ found that parents who had bariatric surgery and perceived their child to have an overweight/obese weight status reported more impaired family functioning. The results of this study, coupled with prior parental reports, indicates that children believe communication is more impaired,^10^ ^,^ ^12^ and this could potentially be due to unhealthy conversations about weight occurring. Future work should assess communication specific to weight and weight loss (sometimes referred to as “weight talk”^10^) to determine if interventions may be needed to aid parent–child dyads with general communication or communication specifically around weight and weight loss.

This is a cross‐sectional analysis, limiting any assumptions of causality. Second, this was a convenience sample, in which every consecutive dyad meeting inclusion criteria was invited to participate. The sample was also similar to other samples limited by homogeneity of White mothers.^9^, ^10^, ^11^, ^12^ The sample also included young children, and the family functioning and communication scales are not consistently reliable with this population. To account for this, supports were provided to young children to help improve the ease of questionnaire completion. Future studies should continue to explore how to best measure family functioning and communication with this younger age group. Finally, the sample size of 23 dyads, with a 43% enrollment rate reflects the challenge with feasibility of obtaining dyadic assessments. Future research should find ways to mitigate the time and travel barriers to parent and child in‐person assessment, such as online or in‐home methods.

Prior research has only assessed perspectives about children from parents in weight management programs, a serious limitation. This study was novel in the enrollment of parents and their children when a parent is initiating a medical weight management program to determine the presenting characteristics of parent–child dyads. Without dyadic assessment, parental underestimation of children's weight control practices and overestimation of sedentary and screen‐time behaviors would not have been observed, and specific child subgroups with overweight/obesity, older children, and males would not have been identified as having less healthy behaviors and impaired family communication. Future research should assess the effects of parental weight loss in weight management programs on perceptions of and changes in actual child weight status and behaviors to provide objective assessments of parental weight loss and weight management program participation effects on children.

## CONFLICT OF INTERESTS

The authors declare that there are no conflict of interests.

## AUTHOR CONTRIBUTIONS

Keeley J. Pratt, ChrisA. Taylor, Colleen Spees, and Sabrena Noria conceived of and received funding for the study. Keeley J. Pratt, Catherine A. VanFossen, Haley M. Kiser, and Colleen Spees carried out the study. Keeley J. Pratt, Catherine A. VanFossen, Haley M. Kiser, and Riley Whiting analyzed the data. All authors were involved in writing the paper and had final approval of the submitted and published manuscript.

## References

[1] Hales CM , Carroll MD , Fryar CD , Ogden CL . Prevalence of Obesity and Severe Obesity Among Adults: United States, 2017–2018. NCHS Data Brief, No 360. Hyattsville, MD: National Center for Health Statistics; 2020. 32487284

[2] Hales CM , Carroll MD , Fryar CD , Ogden CL . Prevalence of Obesity Among Adults and Youth: United States, 2015–2016. NCHS Data Brief, No 288. Hyattsville, MD: National Center for Health Statistics; 2017. 29155689

[3] Snook KR , Hansen AR , Duke CH , et al. Notice of Retraction and Replacement. Snook et al. Change in percentages of adults with overweight or obesity trying to lose weight, 1988‐2014. JAMA. 2018;317(9):971‐973. 10.1001/jama.2016.20036 28267846

[4] Martin CB , Herrick KA , Sarafrazi N , Ogden CL . Attempts to Lose Weight Among Adults in the United States, 2013–2016. NCHS Data Brief, No 313. Hyattsville:National Center for Health Statistics; 2018. 30044214

[5] Agras WS , Hammer LD , McNicholas F , Kraemer HC . Risk factors for childhood overweight: a prospective study from birth to 9.5 years. J Pediatr. 2004;145(1):20‐25. 15238901 10.1016/j.jpeds.2004.03.023

[6] Pratt KJ , Ferriby M , Brown CL , Noria S , Needleman B , Skelton JA . Adult weight management patients’ perceptions of family dynamics and weight status. Clin Obes. 2019;9(5):e12326. 10.1111/cob.12326 31232524 PMC10179550

[7] Pratt KJ , Ferriby M , Noria S , Skelton JA , Taylor CA , Needleman B . Perceived child weight status, family structure and functioning, and support for health behaviors in a sample of bariatric surgery patients. Fam Syst Health. 2020;38(3):300‐309. 10.1037/fsh0000317 29376660

[8] Sellberg F , Ghaderi A , Willmer M , Tynelius P , Berglind D . Change in children’s self‐concept, body‐esteem, and eating attitudes before and 4 years after maternal RYGB. Obes Surg. 2018;28(10):3276‐3283. 29911273 10.1007/s11695-018-3348-z PMC6153582

[9] Brown CL , Pratt KJ , Martin S , Hulshult H , Skelton JA . Weight control practices in children of parents participating in weight management programs. Child Obes. 2019;15(7):451‐458. 31343259 10.1089/chi.2019.0089 PMC6761581

[10] Pratt KJ , Skelton JA , Lewis KH , Taylor CA , Spees C , Brown CL . Family meal practices and weight talk between adult weight management and weight loss surgery patients and their children. J Nutr Educ Behav. 2020;52(6):579‐587. 32527416 10.1016/j.jneb.2020.04.001 PMC10173866

[11] Bureau UC . 2013–2017 American Community Survey 5‐Year Estimates. American Community Survey.; 2018.

[12] Kiser H , Pratt K , Van Fossen C . Brief report: bariatric surgery patients’ communication and engagement in activities with their children. Obes Surg. 2020;30(8):3242‐3246. 32279185 10.1007/s11695-020-04562-9

[13] Adamo KB , Brett KE . Parental perceptions and childhood dietary quality. Matern Child Health J. 2014;18:978‐995. 10.1007/s10995-013-1326-6 23817727

[14] Corder K , van Sluijs EMF , McMinn AM , Ekelund U , Cassidy A , Griffin SJ . Perception versus reality: awareness of physical activity levels of British children. Am J Prev Med. 2010;38(1):1‐8. 10.1016/j.amepre.2009.08.025 20117551 PMC3746297

[15] De Los Reyes A , Augenstein TM , Wang M et al. The validity of the multi‐informant approach to assessing child and adolescent mental health. Psychol Bull. 2015;141(4):858‐900. 25915035 10.1037/a0038498 PMC4486608

[16] Korelitz KE , Garber J . Congruence of parents' and children's perceptions of parenting: a meta‐analysis. J Youth Adolesc. 2016;45(10):1973‐1995. 10.1007/s10964-016-0524-0 27380467 PMC5222679

[17] Mansfield AK , Keitner GI , Dealy J . The family assessment device: an update. Fam Process. 2015;54(1):82‐93. 10.1111/famp.12080 24920469

[18] Pratt KJ , Jalilvand A , Needleman B , Urse K , Ferriby M , Noria S . Postoperative outcomes based on patient participation in a presurgery education and weight management program. Surg Obes Relat Dis. 2018;14(11):1714‐1723. 30274740 10.1016/j.soard.2018.08.006 PMC6752953

[19] Fryar CD , Gu Q , Ogden CL . Anthropometric Reference Data for Children and Adults: United States, 2007‐2010. Vital Health Stat 11. 2012:(252):1‐48. 25204692

[20] Centers for Disease Control and Prevention (CDC) . All About Adult BMI. 2020. https://www.cdc.gov/healthyweight/assessing/bmi/adult_bmi/index.html

[21] Centers for Disease Control and Prevention (CDC) . Growth Charts ‐ Z‐Score Data Files. 2019. https://www.cdc.gov/growthcharts/zscore.htm

[22] Neumark‐Sztainer D , Wall MM , Larson N , et al. Secular trends in weight status and weight‐related attitudes and behaviors in adolescents from 1999 to 2010. Prev Med. 2012;54(1):77‐81. 22024221 10.1016/j.ypmed.2011.10.003 PMC3266744

[23] Ryan CE , Epstein NB , Keitner GI , Miller IW , Bishop DS . Evaluating and Treating Families: The McMaster Approach. New York, NY: Taylor & Francis; 2005.

[24] Hamilton E , Carr A . Systematic review of self‐report family assessment measures. Fam Process. 2015;55(1):16‐20. 10.1111/famp.12200 26582601

[25] Dorsey RR , Eberhardt MS , Ogden CL . Racial/ethnic differences in weight perception. Obesity. 2009;17(4):790‐795. 10.1038/oby.2008.603 19148119

[26] Ling J , Stommel M . Parental and self‐weight perceptions in U.S. Children and adolescents, NHANES 2005‐2014. West J Nurs Res. 2018;41(1):42–57. 10.1177/0193945918758274 29436293

[27] Richmond TK , Thurston I , Sonneville K , Milliren CE , Walls CE , Austin SB . Racial/ethnic differences in accuracy of body mass index reporting in a diverse cohort of young adults. Int J Obes. 2015;39(3):546‐548. 10.1038/ijo.2014.147 PMC4305493 25059116

[28] Stice E , Mazotti L , Krebs Melora , Martin S . Predictors of adolescent dieting behaviors: a longitudinal study. Psychol Addict Behav. 1998;12(3):195‐205. 10.1037/0893-164X.12.3.195

[29] Herzer M , Godiwala N , Hommel KA , et al. Family functioning in the context of pediatric chronic conditions. J Dev Behav Pediatr. 2010;31(1):1‐14. 10.1097/DBP.0b013e3181c7226b 20081433 PMC2821736

[30] Van Fossen CA , Pratt KJ , Murray R , Skelton JA . Family Functioning in Pediatric Primary Care Patients. Clinical Pediatrics. 2018;57 (13):1549–1557. 10.1177/0009922818793347 30095008

